# Supplementary material for: Elucidating the Immune Response to SARS-CoV-2: Natural Infection versus Covaxin/Covishield Vaccination in a South Indian Population
Source: Viruses. 2024 Jul 23;16(8):1178. doi: 10.3390/v16081178 (PMC11360806; doi:10.3390/v16081178)
Supplement: Supplementary file 1 [file viruses-16-01178-s001.zip › viruses-3049542-supplementary.pdf]

Sup. Fig. 1A :Gating Strategy B-cell subsets

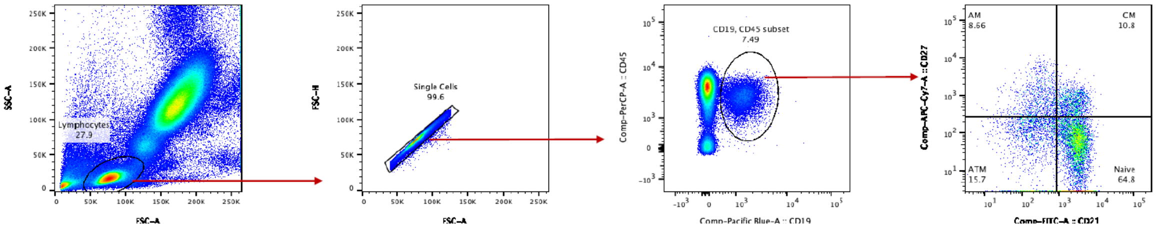

Sup. Fig. 1B: Gating Strategy Memory T-cell subsets

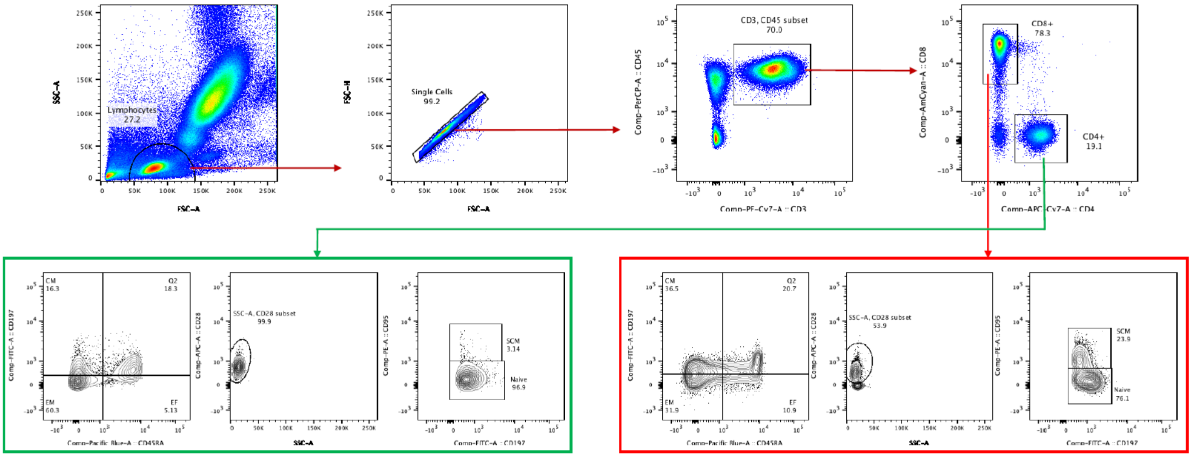

Sup. Fig. 1c: Gating Strategy Monocytes

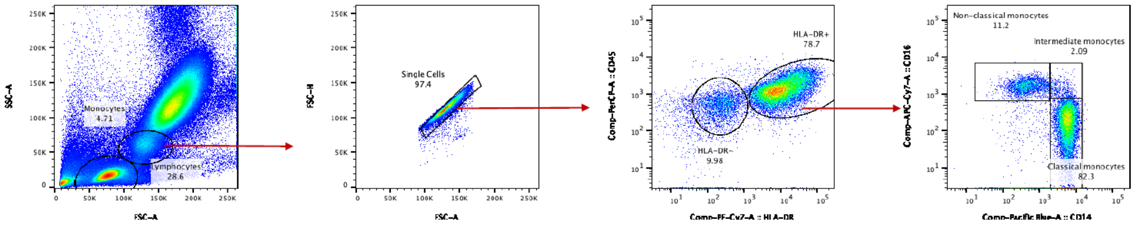

Supplementary Figure 1: Flowcytometry gating strategy for memory B cells, memory T cells and monocyte subsets

Sup. Fig. 2A

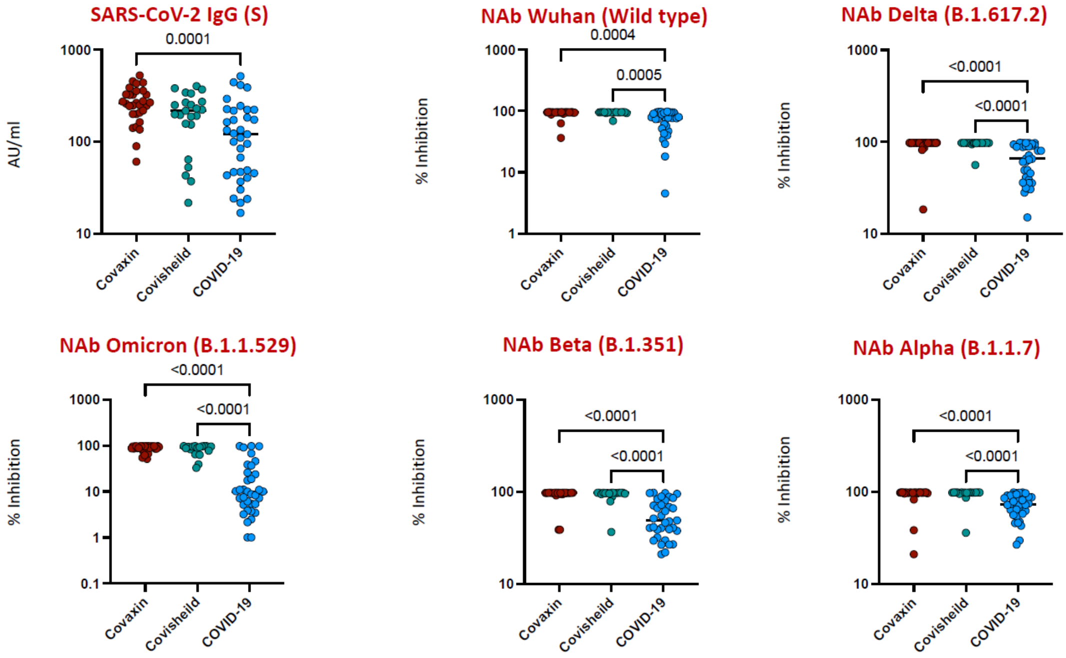

Sup. Fig. 2B: Immune Cell Subsets

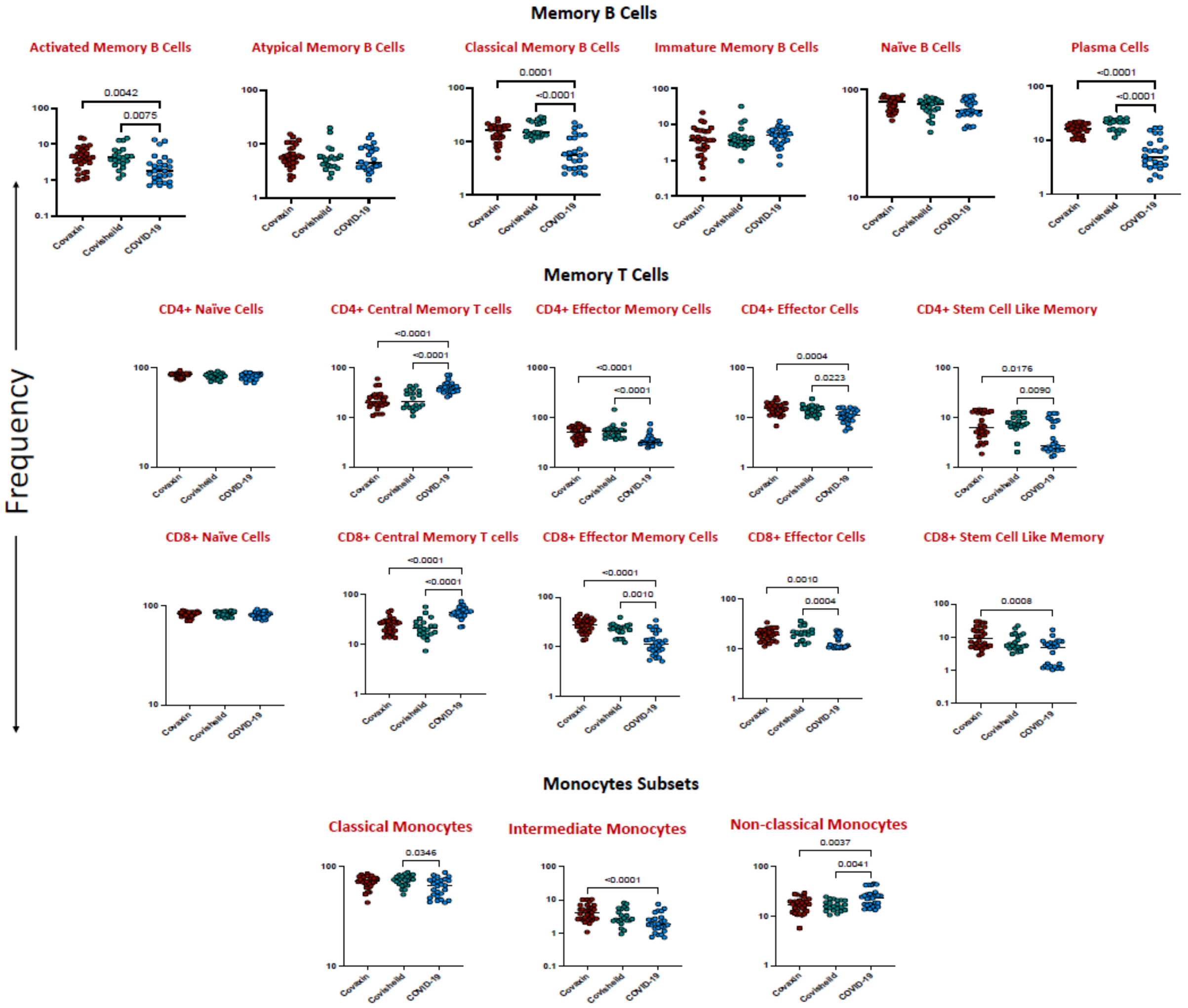

Sup. Fig. 2C: Inflammatory Cytokines

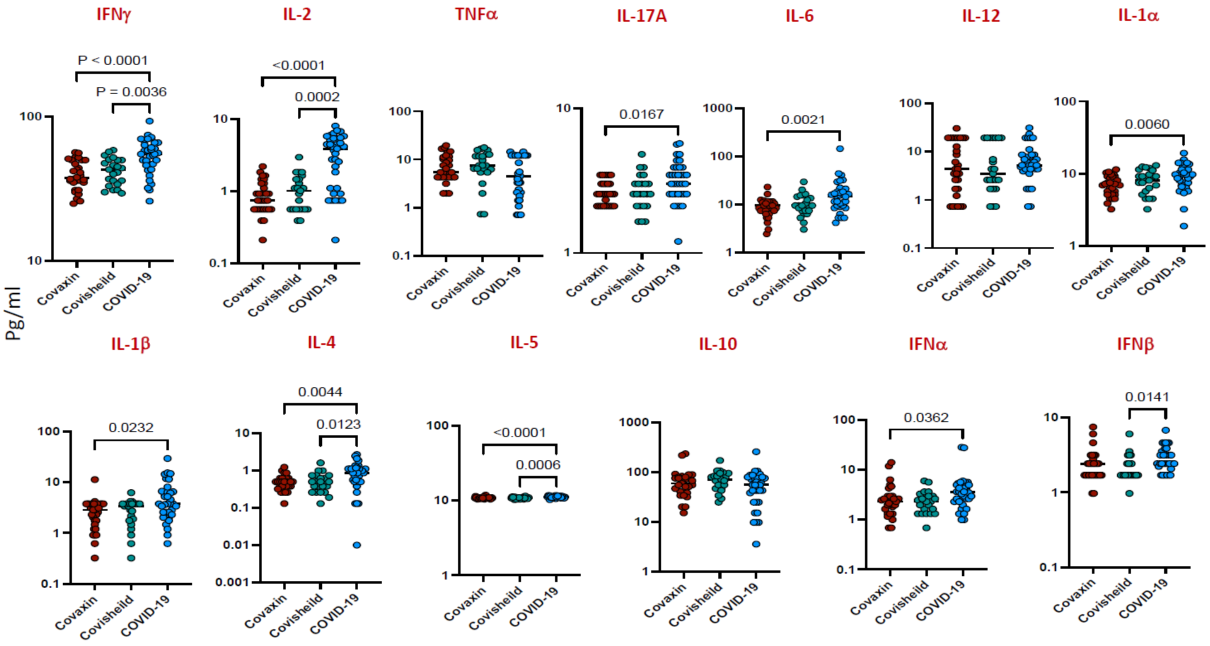

Supplementary Figure 2 : [A] The plasma levels of SARS-CoV2 binding antibodies of IgG [S] and Surrogate Virus Neutralization for wild type and variant lineages of B.1.617.2 (Delta), B.1.1.529 (Omicron), B.1.351 (Beta, SA) and B.1.1.7 (Alpha, UK) in those with Covaxin, Covishield and COVID-19 infection.[B] Frequencies of B cell subsets, T cell subsets and monotytes in those with Covaxin Covishield and COVID-19 infection. [C] Plasma levels a panel of inflammatory cytokine levels are measured in those with Covaxin, Covishield and COVID-19 infection.

Sup. Table 1 : Demographic profile of Covaxin, Covishield and COVID-19 positive cases

Sup. Table 1 :Demographic data of study population				
**S.No**	**CHARACTERISTICS OF STUDY POPULATION**	**COVAXIN(n=30)**	**COVISHIELD(n=23)**	**COVID 19 POSITIVE CASES(n=26)**
1	**Age**	47.5(18-63)	37(21-62)	35(21-58)
2	**Male**	13	12	15
3	**Female**	17	11	11
4	**WBC 10^3/mL**	8.795(4.59-11.1)	7.45(4.47-14.13)	7.65(2.12-10.5)
5	**RBC 10^6/mL**	4.945(4.23-6.11)	4.705(3.13-5.94)	4.93(3.71-7.26)
6	**HGB g/dl**	13.63(9.42-18.06)	13.21(9.48-18.06)	14.165(9.95-19.9)
7	**HCT %**	41.45(31.3-52.8)	40.7(29.9-53.3)	42.2(31.6-59)
8	**PLT 10^3/mL**	284(127.9-392.6)	274.8(203.9-455.1)	241.4(129.8-350.9)
9	**Neutrophil 10^3/mL**	4.39(2.46-6.82)	3.71(2.08-10.68)	3.035(0.76-5.8)
10	**Lymphocyte 10^3/mL**	3.4(1.77-4.98)	2.795(1.98-3.89)	3.095(1.17-5.75)
11	**Monocyte 10^3/mL**	0.46(0.24-0.88)	0.415(0.32-0.63)	0.53(0.02-2.16)
12	**Eosinophil 10^3/mL**	0.2(0.04-0.71)	0.15(0.04-0.51)	0.15(0.05-0.55)
13	**Basophil 10^3/mL**	0.08(0.03-0.15)	0.07(0.03-0.11)	0.05(0-0.1)
14	**Neutrophil %**	52.465(35.01-61.99)	54.31(36.21-76.48)	38.245(20.7-65.47)
15	**Lymphocyte %**	38.075(29.61-54.14)	34.79(19.94-49.76)	42.245(25.99-64.6)
16	**Monocyte%**	5.82(2.89-8.43)	5.675(2.7-8.95)	6.82(1.15-28)
17	**Eosinophil %**	2.22(1.2-6.6)	1.725(0.33-7.37)	1.8(0.7-14.06)
18	**Basophil %**	0.92(0.37-1.84)	0.905(0.36-1.61)	0.645(0-1.8)
19	**MCV fL**	83.05(68.4-102.5)	86.9(72.8-104.1)	85.6(59-94.4)
20	**MCH Pg**	27.9(20.6-34.4)	28.75(22.4-37.7)	28.6(18.7-32.6)
21	**MCHC g/dl**	32.8(30.1-38.2)	32.8(30.8-39.5)	33.3(31.1-34.6)
22	**SARS COV 2 Ig G(S) (AU/ML)**	262.02(60.57-526.62)	225.93(21.68-401.37)	115.285(16.82-514.97)
23	**NAb_Wild type**	95.43(36.38-95.73)	95.48(69.04-97.75)	76.305(4.52-96.77)
24	**NAb_Delta**	97.465(18.47-97.88)	97.515(56.11-97.83)	65.755(28.18-97.93)
25	**NAb_Omicron**	92.205(51.12-97.6)	88.395(0.2-97.39)	8.12(-17.75-97.44)
26	**NAb_SA**	97.215(38.65-97.7)	97.01(13.3-97.65)	48.375(20.9-97.6)
27	**NAb_UK**	97.99(20.95-98.27)	98.05(35.89-98.19)	72.05(29.55-98.19)

Sup. Table 2 : Geometric mean of Immune cell types in those with Vaccinated, COVID-19 infection and Healthy controls

Sup. Table 2

Immune Cell Types	Vaccinated	COVID-19	Histroric Healthy Controls
	Geo Mean		
Activated Memory B Cells	3.995	2.029	1.223
Classical Memory B Cells	15.14	6.025	3.221
Plasma Cells	17.45	5.511	3.933
CD4+ Central Memory T cells	21.52	39.46	15.33
CD4+ Effector Memory T cells	49.67	34.28	17.82
CD4+ Effector cells	14.55	10.75	7.43
CD4+ Stem Cell like Memory	7.068	3.999	2.93
CD8+ Central Memory T cells	22.65	43.21	16.543
CD8+ Effector Memory T cells	24.64	11.52	8.443
CD8+ Effector cells	19.39	13.69	8.933
CD8+ Stem Cell like Memory	8.494	3.366	2.554
Classical Monocytes	70.15	61.94	50.23
Intemediate Monocytes	3.561	1.956	1.22
Non-Classical Monocytes	16.09	23.26	11.22
IFNg	40.04	52.81	20.22
IL-2	0.8612	2.621	0.554
IL-17A	2.639	3.037	1.22
IL-6	8.994	14.69	5.332
IL-1a	7.142	8.749	5.422
IL-1b	2.34	3.612	1.882
IL-4	0.4619	0.6626	0.432
IL-5	10.82	11.15	7.22
IFNa	2.278	3.418	1.22
IFNb	2.181	2.775	1.43

Sup. Table 2 : Geometric mean of the representing immune parameters measured among the Vaccinated, COVID-19 with references to the historic healthy controls who were non-infected and non-vaccinated.

Sup. Table 3

Inflammatory Markers	COVID-19 Asymptomatic	COVID-19 mild and moderate	pValue
IFNg	55.3	50.29	0.3063
IL-2	3.617	1.864	0.268
TNFa	3.325	5.843	0.1449
IL-17A	3.191	2.899	0.639
IL-6	16.35	13.02	0.3167
IL-12	4.42	7.664	0.062
IL-1a	8.951	8.563	0.8255
IL-1b	3.712	3.51	0.9545
IL-4	0.8261	0.5381	0.3203
IL-5	11.17	11.12	0.9146
IL-10	39.51	47.95	0.3717
IFNa	3.327	3.523	0.6758
IFNb	2.797	2.754	>0.9999

Sup. Table 3 : Geometric mean of plasma levels; a panel of inflammatory cytokine levels measured in the COVID-19 asymptomatic and COVID-19 mild and moderate cases.
